# Air Pollution and the Novel Covid-19 Disease: a Putative Disease Risk Factor

**DOI:** 10.1007/s42399-020-00274-4

**Published:** 2020-04-15

**Authors:** Luigi Martelletti, Paolo Martelletti

**Affiliations:** 1 MSc in Energy and Environmental Technology and Economics, City University of London, 6 Tibberton Square, London, N1 8SF UK; 2 Emergency Medicine CoViD-19 Unit, Sant’Andrea University Hospital, Sapienza University, Rome, Italy

The emergence of a new Severe Acute Respiratory Syndrome coronavirus (SARS-CoV-2) disease (Covid-19) appeared in Wuhan (China) in December 2019 when the Chinese authorities reported to the World Health Organization (WHO) Course Office in China of a pneumonia of unknown cases. Consequently, the novel coronavirus outbreak was firstly declared as a Public Health Emergency of International Concern on 30 January and then it was officially confirmed as a Pandemic on 11 February [1].

The severity of this virus lies in its long incubation period, which it is reported to range up to 14 days (although it has been recently stated that, in some cases, this may extend to 21 days [2]), and the respiratory illness related to the virus, which can develop from a common cold to a more deadly disease such as SARS and MERS (Middle East Respiratory Syndrome) [3]. According to the scientific information released by the CDC (Centre for Disease Control and Prevention) regarding the symptoms of Covid-19, the pathologies this virus develops and presents range from 2 to 14 days. The infections caused by the virus vary and can range from being mild (i.e., not showing any symptoms) to more severe, which in some cases can lead to hospitalization. Symptoms include fever, which is the most common, followed by dry cough, and shortness of breath [4]. The manifestation of these symptoms can depend on the patient’s age and physical condition. Older people, those who live in a nursing home or long-term care facilities, and all people with existing health conditions are those who will develop more critical illness [5].

There is the additional environmental aspect, which could facilitate the spread of the virus, namely the high agglomeration of air pollutants. The assumption that air pollution conditions facilitate the spread of the virus was shown and supported by Cui et al. [6] during the SARS outbreak in mainland China in November 2002. This study analyzed the correlation between the increment of the API (Air Pollution Index) and the rate of fatality due to SARS across 5 regions in China. The regions were selected according to their elevated Air Pollution Index, taking into consideration that an API less than 100 is thought to be healthy for the general population. According to this research, the five regions under investigation (Guangdong, Shanxi, Hebei, Beijing, and Tianjin) presented a linear relationship between API, in the period April to May 2003, and fatality rate due to SARS. The lower the API, the lower the mortality rate [6].

In 2017, Ciencewicki and Jaspers conducted an epidemiological analysis regarding air pollution and respiratory viral infections which noted positive correlation between the high level of particulate matter (PM) in some urban areas and mortality due to cardiovascular and respiratory conditions. Elevated exposure to common PM present in the air can alter host immunity to respiratory viral infections [7].

**Fig. 1:**
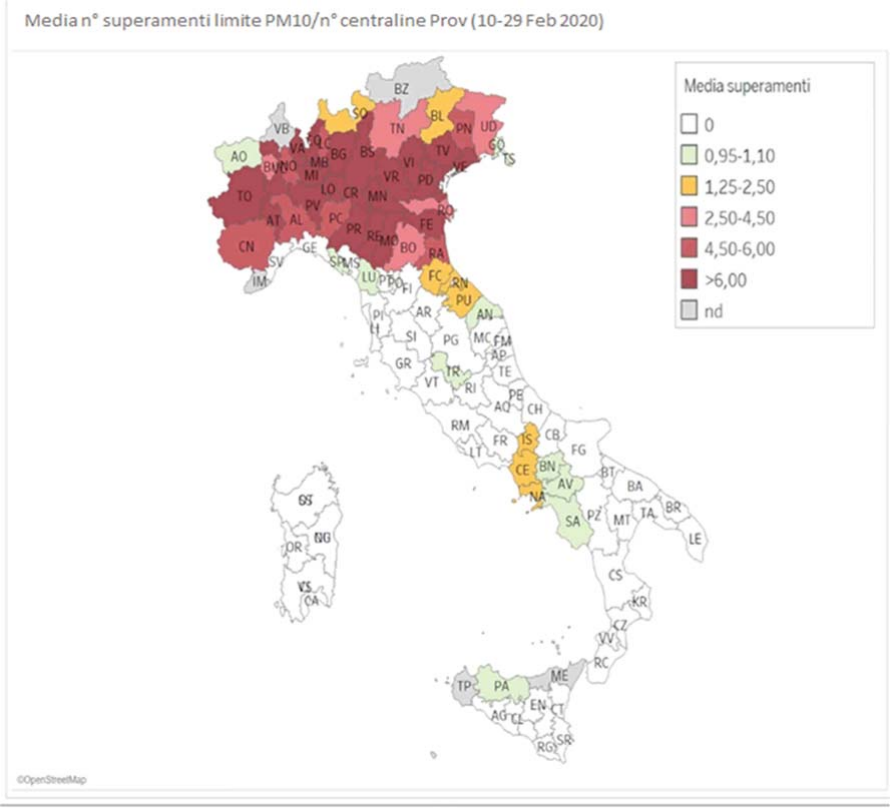
Particulate matter emissions drop over Italy in February 2020 [8]

**Fig. 2:**
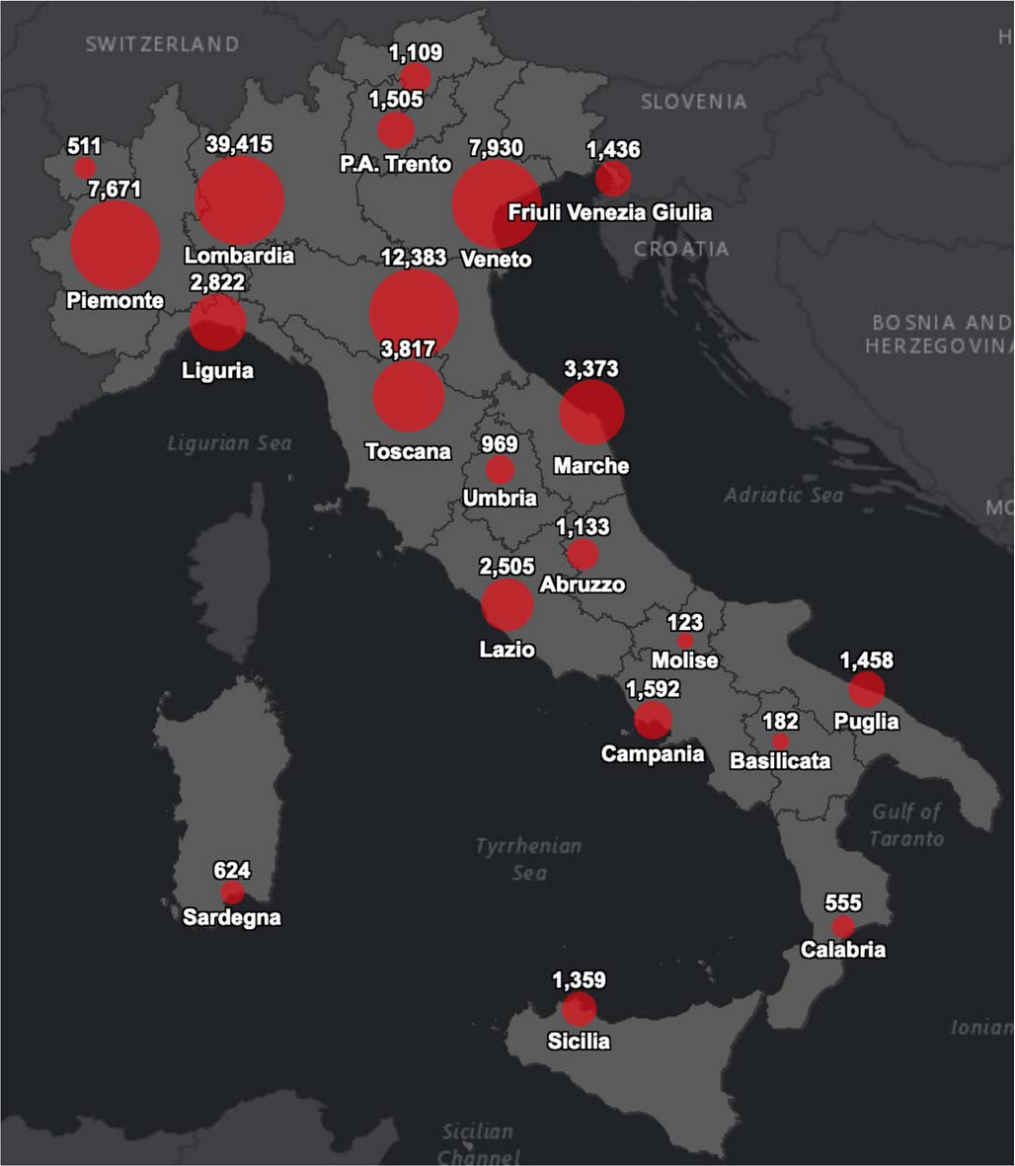
CoViD-19 cases over Italy [9]

A recent study from the SIMA (Società Italiana di Medicina Ambientale) reported that the specificity of the high spread of the contagious virus in some areas of Northern Italy is likely to be linked to air pollution conditions. According to the recent SIMA analysis of Covid-19 diffusion in Italy, the atmospheric particulate matter exercises a *carrier* (or *boost*) action along with the virus. The PM_10_ (particulate matter) is composed of solid and liquid particles which allow to float in the airflow longer and to be widespread over larger distances. Atmospheric PM has a sub-layer that facilitates the virus survival in airflows for hours or days. The local atmospheric aspect is another environmental factor that must be considered in the accelerated diffusion of this virus. In fact, SARS-CoV-2 has facilitated activation rates when in presence of high local relative humidity, while it is inhibited in hot climate situations [8]. This research shows how the Italian Northern Regions, which have been the most affected by Covid-19, are also those with a high amount of atmospheric particulate matter (PM_10_ and PM_2.5_) going above the legislative standards (limit, 50 μg/m^3^ per day) in the month of February 2020. This relationship can also be seen by comparing the following two images which illustrate the nitrogen dioxide emissions and the Covid-19 case fatality in Northern Italy during January 2020. The red zone in Fig. 1 indicates high PM agglomerations while the red circles in Fig. 2 designate those infected. A correlation between elevated concentration of PM and the high spread and mortality rate is visible.

**Fig. 3:**
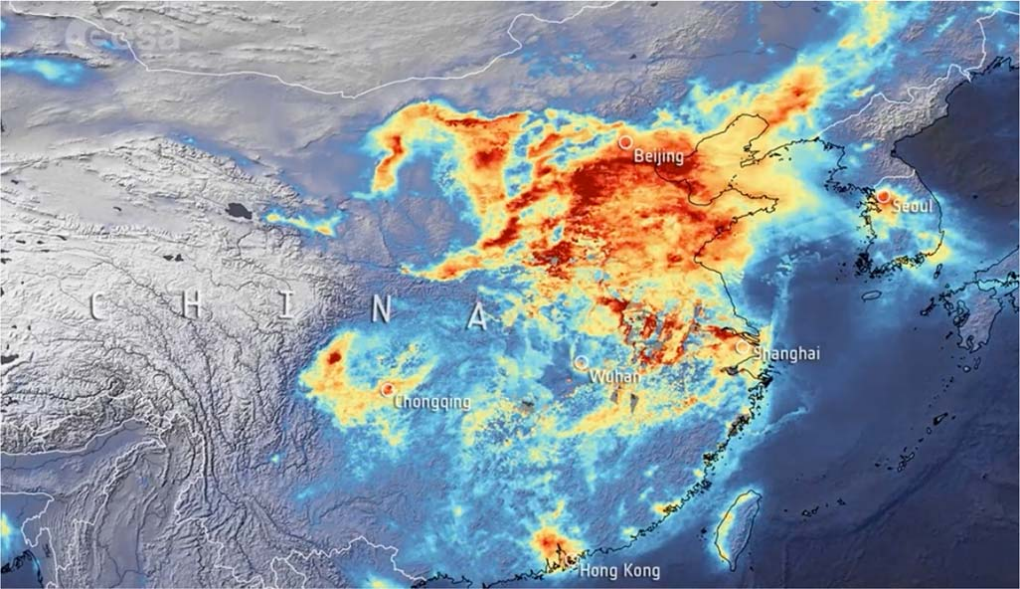
Nitrogen dioxide emissions in mainland China [10]

**Fig. 4:**
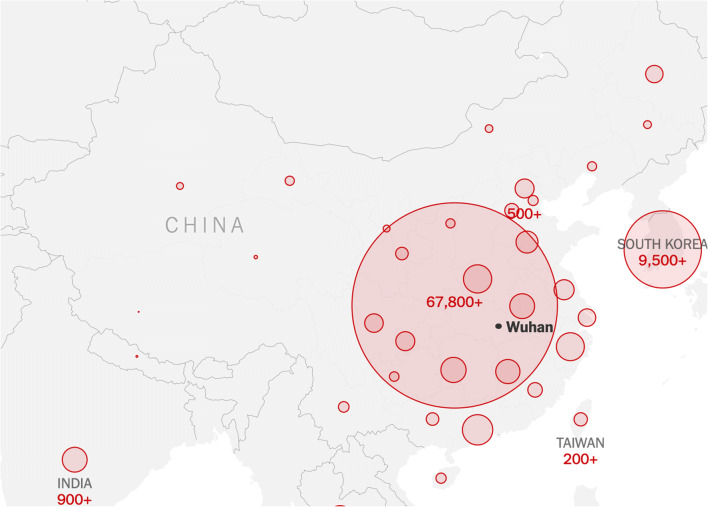
Mainland China and CoViD-19 cases [11]

Although the connection can be considered both a false statement as it lacks data and causality, China has faced a near identical situation with the epicenter of the Covid-19 pandemic, where elevated concentrations of air pollutants were present in the regions mostly affected (see Figs. 3 and 4). The above studies show that air pollutants, such as particulate matter, nitrogen dioxide, and carbon monoxide, are most likely direct to facilitate the longevity of virus particles in favorable climate conditions.

SARS virus and other respiratory diseases such as COPD (chronic obstructive pulmonary disease) find fertile “territory” in air pollutant particles and, in a linear relationship, they survive longer and become more aggressive in an immune system already aggravated by these harmful substances. This hypothesis needs to be validated by further future epidemiological studies in multiple geographic regions affected by the Covid-19 pandemic.
